# Substance use and use disorders among Veterans on long-term opioid therapy

**DOI:** 10.1016/j.dadr.2025.100347

**Published:** 2025-05-19

**Authors:** Thye Peng Ngo, Salomeh Keyhani, Samuel Leonard, Katherine J. Hoggatt

**Affiliations:** a Center for Data to Discovery and Delivery Innovation (3DI), San Francisco VA Health Care System, CA, USA; b National Clinician Scholars Program (NCSP) at UCSF, San Francisco, CA, USA; c School of Medicine, University of California, San Francisco, CA, USA

**Keywords:** Veterans, Long-term opioid therapy, Substance co-use, Mortality, Fatal overdose, Traumatic deaths

## Abstract

**Background:**

Few studies have reported on the prevalence and health risks associated with substance use and substance use disorder (SU/SUD) in Veterans who use long-term opioid therapy (LTOT). We leveraged health record data to estimate SU/SUD prevalence and its association with mortality among Veterans on LTOT.

**Methods:**

We conducted a secondary analysis of cohort data for Veterans on LTOT within Veterans Health Administration outpatient settings (2014–2019). SU/SUD was defined as a positive screen for risky alcohol use; a positive urine drug screen for cannabis, benzodiazepines, or stimulants; or a documented SUD diagnosis. We fit Cox models for all-cause mortality, fatal overdose, and traumatic deaths, comparing Veterans on LTOT with SU/SUD vs. LTOT-only.

**Results:**

One in four (25.0 %) Veterans on LTOT have risky alcohol use, tested positive for other substances, or had a diagnosed SUD. Alcohol was the most common SU/SUD (9.8 %), followed by sedative (8.1 %), cannabis (6.6 %), and stimulant (0.6 %). Relative to Veterans on LTOT only, mortality rates were higher for Veterans on LTOT with cannabis (HR=1.16, 95 % CI=1.03, 1.30), sedative (HR=1.29, 95 % CI=1.10, 1.52), or stimulant SU/SUD (HR=1.54, 95 % CI=1.17, 2.02). Fatal overdose rates were higher for LTOT with alcohol (HR=1.43, 95 % CI=1.10, 1.86), sedatives (HR=1.40, 95 % CI=1.04, 1.91), or stimulant SU/SUD (HR=3.29, 95 % CI=1.60, 6.77). LTOT with sedative SU/SUD was associated with traumatic death rates (HR=1.30, 95 % CI=1.05, 1.61).

**Conclusion:**

Substance co-use is common among Veterans on LTOT and is associated with elevated mortality and overdose risks. Comprehensive screening and targeted interventions may be needed.

## Highlights

• 1 in 4 Veterans on LTOT engage in substance use (SU) or have a diagnosed SUD.
• Alcohol is the most common SU/SUD, followed by sedatives and cannabis.
• Cannabis, sedative, or stimulant SU/SUD is associated with higher all-cause mortality.
• Sedative SU/SUD is associated with increased fatal overdose and traumatic death.
• Stimulant SU/SUD triples fatal overdose rates among Veterans on LTOT.

## 1 Introduction

Substance use disorder (SUD) is pervasive in the U.S., affecting over 48 million Americans aged 12 and above in 2022 (Substance Abuse and Mental Health Services Administration, 2023). The impact of SUD on healthcare systems and society is immense, with opioids and stimulants accounting for 81.8 % and 57.1 % of drug overdose deaths, respectively in 2022 (Centers for Disease Control and Prevention, 2023). SUD-related healthcare costs are estimated at $35 billion, including $10.2 billion for alcohol-related disorders and $7.3 billion for opioid-related disorders (Li et al., 2023). SUD also contributes to increased hospitalizations and emergency department visits (Lewer et al., 2020), incurring significant costs for healthcare system.

Veterans represent a particularly vulnerable subpopulation for opioid misuse. Veterans have high rates of chronic pain, often due to service-related injuries and trauma, and many rely on opioid therapy for pain management. Despite reductions in opioid prescriptions over the past decade, more than 50 % of Veterans prescribed opioids in 2023 were on long-term opioid therapy (LTOT) (U.S. Department of Veterans Affairs, 2023), which is using opioids for pain for more than three months (Karmali et al., 2020). Many Veterans initiated on LTOT maintain persistently high opioid use for over two years (Hayes et al., 2021), increasing their risk for opioid use disorder (OUD). In addition to chronic pain, many Veterans experience high rates of PTSD and depression (Athey and Overholser, 2018, Lan et al., 2016), as well as socioeconomic vulnerabilities, such as housing instability and financial insecurity (Austin et al., 2021), which also increase the risk for opioid and other SUD. Overall,18 % of Veterans diagnosed with SUD in the past year in 2022, with one study revealed 58.8 % of Veterans diagnosed with OUD are also diagnosed with at least one additional SUD (Lin, Bohnert, et al., 2021).

The health risks associated with LTOT may be greater for the many patients who use other substances (Dobscha et al., 2013, Vowles et al., 2022). Furthermore, co-use of other substances, such as sedatives, in addition to LTOT, have been shown to increase risk adverse health outcomes, including falls and emergency department visits (Yarborough et al., 2019). Current opioid prescribing guidelines primarily address risks associated with opioid co-use with alcohol and sedatives (e.g., benzodiazepines) and pay less attention to co-use with other substances, such as cannabis and stimulants (Dowell et al., 2022, VA/DoD Clinical Practice Guideline, T. L. G., 2022). This oversight is concerning, as stimulants and other substances were detected in 63 % of opioid-related overdose deaths across 25 U.S. states (Gladden et al., 2019). Additionally, overdose mortality involving cocaine and psychostimulants (e.g., methamphetamine) alone among Veterans increased by over 200 % and 600 %, respectively, from 2010 to 2019 (Begley et al., 2022).

To date, comprehensive data on the prevalence and mortality risks of SU/SUD among Veterans on LTOT remains limited. While prior research has identified risk factors for multiple SU/SUD in Veterans (Bhalla et al., 2017, Cypel et al., 2023), explored overdose mortality trends (Begley et al., 2022), and examined the association between SUDs and suicide mortality (Bohnert et al., 2017), these studies do not focus on Veterans receiving LTOT with other forms of substance use (i.e., multiple SU/SUDs) using biomarker data. In addition, existing studies examining substance co-use with LTOT have largely relied on self-reported data or medical records without biomarker confirmation (e.g., urine drug screening), which may underestimate the true prevalence of substance use.

This study addressed these gaps by leveraging longitudinal data from Veterans Health Administration, incorporating urine drug screens to provide biomarker confirmation of substance use. This study specifically aimed to examine Veterans on LTOT to estimate the prevalence of co-occurring SU/SUD and to assess whether SU/SUD was associated with increased rates of all-cause or cause-specific mortality (fatal overdose and traumatic deaths).

## 2 Methods

### 2.1 Sample

This is a secondary analysis of national VA and Medicare datasets from a retrospective cohort study on the associations of cannabis use with mortality and hospital visits among Veterans on LTOT (Keyhani et al., 2022a). The cohort included all Veterans who completed their first urine drug screening (UDS) in a VA outpatient setting between January 1, 2014, and December 31, 2019 and who were prescribed LTOT (defined as those receiving opioid therapy for at least 84 days within a 90-day period, aligning with typical VA prescribing practices of 30-day dispensing intervals and accounting for refill delays or prescription gaps (Karmali et al., 2020)). The date of the UDS was the “index date” to anchor the assessment of other variables and indicate the start of follow-up for mortality outcomes. Patients were excluded if they were potentially at the end of life (e.g., those residing in a VA community living center, receiving palliative care, or undergoing inpatient cancer chemotherapy) or had a Care Assessment Needs (CAN) score of 99, indicating a very high likelihood of death within 90 days (Wang et al., 2013). We also excluded patients prescribed THC-containing medications (e.g., dronabinol) to avoid false-positive urine drug screens for THC and individuals treated in opiate treatment programs (e.g., methadone clinics) as their primary reason for receiving opioids was opioid use disorder, not pain management. We further excluded opioids formulations inconsistent with chronic pain treatment, including single-agent and combination products primarily used for palliative care or opioid use disorder (e.g., buprenorphine) (Supplemental). However, we did not exclude individuals prescribed stimulants or benzodiazepines, as the study aimed to examine their potential associations with the outcomes. Data were obtained from the VA Corporate Data Warehouse, which contains comprehensive patient demographic information, health-related records, and diagnostic codes, with additional data on Medicare utilized by Veterans in the cohort. The study was approved by institutional review board at the University of California, San Francisco.

### 2.2 Exposure

The primary exposures were for co-use of alcohol, cannabis, stimulants (cocaine and amphetamine), and sedatives (primarily benzodiazepine) along with LTOT. At the VA, urine drug screening (UDS) has been implemented as a risk mitigation strategy for patients prescribed opioids for pain. Engagement with this screening has been monitored since 2013 and became mandatory in 2014. Currently, over 90 % of patients receiving long-term opioids for chronic pain undergo UDS (Sandbrink et al., 2020). Risky alcohol use (consumption above recommended limits) was measured using the 3-item Alcohol Use Disorder Identification Test (AUDIT-C), with a cut-off score of 4 for men and 3 for women (Bradley et al., 1998, Bush et al., 1998). Opioids, cannabis, stimulant, and benzodiazepine use was detected by at least one positive UDS. Substance-specific use disorders were measured based on the International Classification of Diseases (ICD) 9 and 10 codes for alcohol, cannabis, stimulant, and sedative use disorders within the prior two years before the index date (Supplemental).

We categorized Veterans on LTOT into five groups, focusing specifically on those with LTOT and one additional SU/SUD. These groups were: (1) LTOT-only as the reference group (without positive UDS for other substance or a ICD-recorded SUD diagnosis) (2) alcohol group (risky alcohol use or ICD-recorded alcohol use disorder), (3) cannabis group (positive cannabis use on UDS or ICD-recorded cannabis use disorder), (4) sedative group (positive benzodiazepine on UDS or ICD-recorded sedative use disorder), and (5) stimulant group (positive stimulant use on UDS or ICD-recorded stimulant use disorders). The sedative group captures primarily benzodiazepines and may include barbiturates.

### 2.3 Outcomes

The outcomes were all-cause mortality, fatal overdose (either intentional, unintentional, or provisional drug-related deaths), and traumatic deaths (accidents or unintentional injuries, suicide, and homicide). These outcomes were extracted from the VA Mortality Data repository, and ICD-9 and 10 codes were used to categorize data related to causes of death (Supplemental).

### 2.4 Covariates

Demographic variables included age, sex, race, ethnicity, and marital status (Table 1). Health-related factors comprised mental health conditions (psychosis, depression, bipolar disorder, posttraumatic stress disorder, anxiety, and self-harm) and pain-related diagnoses (back and spine disorders, neck and spine disorders, osteoarthritis, neuropathy, headache, and traumatic brain injury). These conditions were identified using the ICD-9 and 10 codes. Social factors were housing insecurity and lack of social support, which both were also confirmed with ICD codes. The ICD-coded health and social factors were assessed for the two years prior to the index UDS. The Care Assessment Need (CAN) score integrates patient demographics, comorbidities, healthcare utilization, and socioeconomic factors to predict hospitalization and estimate 90-day mortality risk (Fihn and Box, 2013, Fihn and Box, 2016). The score ranges from 0 (low) to 99 (high mortality risk), updated weekly, and patients with a score of 99 were excluded.

**Table 1:** Baseline characteristics of Veterans on long term opioid therapy (LTOT, ≥ 84 Days) in past 90 days (N = 167,547).

**Characteristics**		**Total Sample (N = 167,547)** **N (%) or Mean (SD, range)**	**With Any Other SU/SUD (N = 128,185)** **N (%) or Mean (SD)**		**Without Other SU/SUD (N = 39,362)** **N (%) or Mean (SD)**	**P-value**
**Demographics**						
Age		62.7 (11.4, 21–117)	60.5 (11.7)		60.5 (10.2)	< 0.001
18–34		3307 (2.0 %)	978 (2.5 %)		2329 (1.8 %)	< 0.001
35–49		14,951 (8.9 %)	3670 (9.3 %)		11,281 (8.8 %)	
50–64		60,290 (36.0 %)	17,492 (44.4 %)		42,798 (33.4 %)	
65–74		67,375 (40.2 %)	15,109 (38.4 %)		52,266 (40.8 %)	
75 or older		21,624 (12.9 %)	2113 (5.4 %)		19,511 (15.2 %)	
Sex						
Male		157,026 (93.7 %)	37,438 (95.1 %)		119,588 (93.3 %)	< 0.001
Female		10,521 (6.3 %)	1924 (4.9 %)		8597 (6.7 %)	
Race						
American Indian or Alaska Native		1729 (1.0 %)	512 (1.3 %)		1217 (1.0 %)	< 0.001
Asian		396 (0.2 %)	87 (0.2 %)		309 (0.2 %)	
Black or African American		19,854 (11.9 %)	5231 (13.3 %)		14,623 (11.4 %)	
Native Hawaiian or Other Pacific Islander		1168 (0.7 %)	281 (0.7 %)		887 (0.7 %)	
White		134,282 (80.2 %)	30,776 (78.2 %)		103,506 (80.8 %)	
More than one race/missing		10,118 (6.0 %)	2475 (6.3 %)		7643 (6.0 %)	
Hispanic/Latinx		5628 (3.4 %)	1539 (3.9 %)		4089 (3.2 %)	< 0.001
Married		88,931 (53.1 %)	16,795 (42.7 %)		72,136 (56.3 %)	< 0.001
**Health-Related Factors**						
Mental health diagnoses^a^ (prior 2 years)						
Psychosis		4209 (2.5 %)	1457 (3.7 %)		2752 (2.2 %)	< 0.001
Depression		60,435 (36.1 %)	17,221 (43.8 %)		43,214 (33.7 %)	< 0.001
Bipolar		6391 (3.8 %)	2418 (6.1 %)		3973 (3.1 %)	< 0.001
PTSD^b^		17,481 (10.4 %)	5469 (13.9 %)		12,012 (9.4 %)	< 0.001
Anxiety		34,010 (20.3 %)	9930 (25.2 %)		24,080 (18.8 %)	< 0.001
Self-harm		572 (0.3 %)	301 (0.8 %)		271 (0.2 %)	< 0.001
Pain-related diagnoses^a^ (prior 2 years)						
Back and spine disorders		117,679 (70.2 %)	27,638 (70.2 %)		90,041 (70.2 %)	0.915
Neck and spine disorders		39,476 (23.6 %)	9770 (24.8 %)		29,706 (21.2 %)	< 0.001
Osteoarthritis		57,273 (34.2 %)	12,654 (32.2 %)		44,619 (34.8 %)	< 0.001
Neuropathy		33,909 (20.2 %)	6792 (17.3 %)		27,117 (21.2 %)	< 0.001
Headache		16,903 (10.1 %)	3936 (10.0 %)		12,967 (10.1 %)	0.503
Traumatic brain injury		225 (0.1 %)	71 (0.2 %)		154 (0.1 %)	0.004
Care Assessment Needs (CAN) score		51.4 (27.4, 0–99)	60.5 (10.2)		63.4 (11.7)	< 0.001
Hospitalization in the past year		28,969 (17.3 %)	7824 (19.9 %)		21,145 (16.5 %)	< 0.001
**Social-Related Factors**						
Homelessness and marginally housed^a^ (prior 2 years)		7803 (4.7 %)	3374 (4.7 %)		4429 (3.5 %)	< 0.001
Lack of social support^a^ (prior 2 years)		1575 (0.9 %)	556 (1.4 %)		1019 (0.8 %)	< 0.001
**Substance-Related Conditions**						
Substance use disorders^a^ (prior 2 years)						
Alcohol use disorder		18,601 (11.1 %)	18,601 (47.3 %)		0 (0.0 %)	< 0.001
Cannabis use disorder		7163 (4.3 %)	7163 (18.2 %)		0 (0.0 %)	< 0.001
Cocaine use disorder		2314 (1.4 %)	1655 (4.2 %)		659 (0.5 %)	< 0.001
Opioid use disorder		12,373 (7.4 %)	4363 (11.1 %)		8010 (6.3 %)	< 0.001
Sedative use disorder		1212 (0.7 %)	843 (2.1 %)		369 (0.3 %)	< 0.001
Stimulant use disorder		917 (0.5 %)	917 (2.3 %)		0 (0.0 %)	< 0.001
Other drug use disorder		6095 (3.6 %)	3292 (8.4 %)		2803 (2.2 %)	< 0.001
Substance use, self-reported (past year)						
Elevated AUDIT-C score (≥ 3 for women and ≥ 4 for men)		6557 (3.9 %)	6.557 (16.7 %)		0 (0.0 %)	< 0.001
Substance use, UDS-screened (past year)						
Positive amphetamine		1305 (0.8 %)	1305 (3.3 %)		0 (0.0 %)	< 0.001
Positive benzodiazepine		17,112 (10.2 %)	6947 (17.7 %)		10,165 (7.93 %)	< 0.001
Positive cannabis		13,870 (8.3 %)	13,870 (35.2 %)		0 (0.0 %)	< 0.001
Positive cocaine		787 (0.5 %)	787 (2.0 %)		0 (0.0 %)	< 0.001
Psychoactive drugs (past 90 days)						
Morphine milligram-equivalents		224.5 (410.3, 0–20,400)	230.9 (2.2)		222.5 (1.1)	< 0.001
Long-acting opioid		49,718 (29.7 %)	11,148 (28.3 %)		38,570 (30.1 %)	< 0.001
Benzodiazepine		30,920 (18.5 %)	7054 (17.9 %)		23,866 (18.6 %)	0.002
GABA drug^c^		50,347 (30.1 %)	11,268 (28.6 %)		39,079 (30.5 %)	< 0.001
Muscle relaxant		35,859 (21.4 %)	8397 (21.3 %)		27,462 (21.4 %)	0.700
Antidepressant		57,312 (34.2 %)	14,236 (36.2 %)		43,076 (33.6 %)	< 0.001
Antipsychotic		12,269 (7.3 %)	3971 (10.1 %)		8298 (6.5 %)	< 0.001
Sedatives		13,145 (7.6 %)	2987 (7.6 %)		10,158 (7.9 %)	0.030
Smoking status						
Current tobacco use		75,350 (45.0 %)	23,004 (58.4 %)		52,346 (40.8 %)	< 0.001
History of tobacco use		36,735 (21.9 %)	7046 (17.9 %)		29,689 (23.2 %)	
No history of tobacco use		35,589 (21.2 %)	5597 (14.2 %)		29,992 (23.4 %)	
Unknown status		19,873 (11.9 %)	1272 (3.2 %)		5329 (4.2 %)	

a Diagnosed based on ICD-9 and ICD-10 codes

b Posttraumatic stress disorder

c γ-aminobutyric acid

### 2.5 Statistical analysis

We assessed differences in the distribution of all-cause mortality and other causes of death across the different SU/SUD groups using chi-square tests for categorical variables and t-tests or Wilcoxon rank-sum tests for continuous variables, as appropriate. We estimated the association between SU/SUD and all-cause mortality using the Cox proportional hazards model. Since the proportional hazards assumption was violated, time-varying covariates were included in the model. For fatal overdose and traumatic deaths, we used the Fine and Gray’s (1999) sub-distribution hazards model to account for competing risks (i.e., all-cause mortality not related to the primary event). All models included demographic covariates, smoking status, psychoactive drugs, CAN score, and hospitalizations within one year, in addition to variables for the different exposure groups. Hazard ratios (HR) and 95 % confidence intervals (CIs) were reported for each outcome. All analyses were conducted using STATA, v18.

## 3 Results

### 3.1 Baseline characteristics

A total of 167,547 Veterans were included in the study. The average age of Veterans on LTOT was 62.7 years (ranged from 21 to 117), predominantly male (93.7 %) and White (80.2 %) (Table 1). Over half were married (53.1 %). Common mental health diagnoses were depression (36.1 %), anxiety (20.3 %), and PTSD (10.4 %). More than two-thirds were diagnosed with back and spine disorders (70.2 %), followed by osteoarthritis (34.2 %) and neck and spine disorders (23.6 %).

Alcohol use disorder was the most prevalent substance-specific disorder in Veterans on LTOT (11.1 %), followed by opioid use disorder (7.4 %), cannabis use disorder (4.3 %), cocaine use disorder (1.4 %), sedative use disorder (0.7 %), and stimulant use disorder (0.5 %). About 3.9 % of Veterans on LTOT had risky alcohol use (based on AUDIT-C scores), 10.2 % of them had UDS-positive benzodiazepine use, and 8.3 % had UDS-positive cannabis use.

### 3.2 Combination of multiple substance use and disorders

**Fig. 1:**
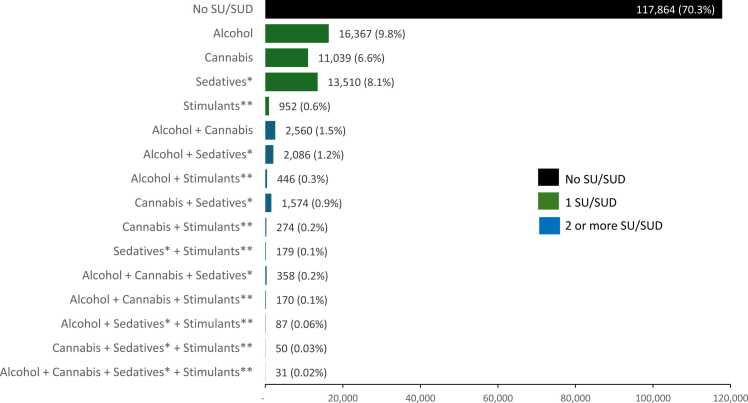
Combination of Substance Use/Substance Use Disorders (SU/SUD) among Veterans on Long Term Opioid Therapy (LTOT) (N = 167,547) Note: SU/SUD classification was based on ICD-9 and ICD-10 diagnoses within the past two years, positive urine drug screens (UDS) in the past year, and self-reported AUDIT-C scores in the past year.⁎Sedative SU/SUD include positive benzodiazepine on UDS and ICD-recorded sedative use disorder.⁎⁎Stimulant SU/SUD include positive stimulant on UDS and ICD-coded stimulant use disorder.

**Fig. 2:**
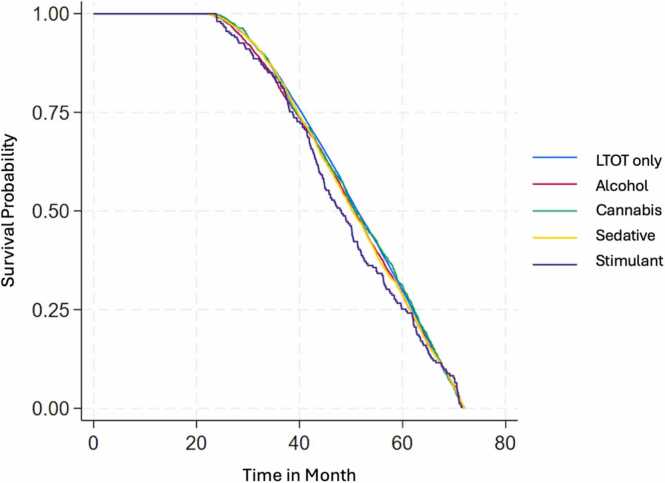

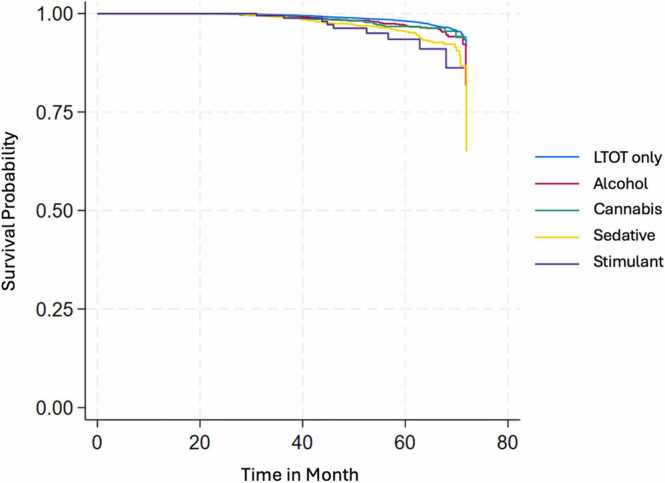

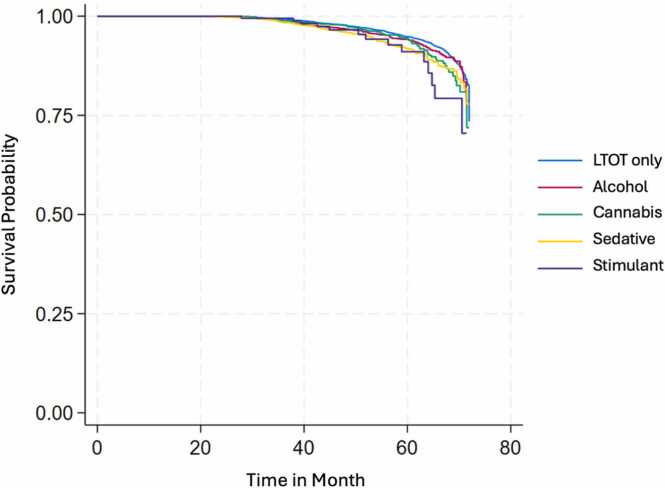
a Survival Estimates for All-Cause Mortality by Substance Use and Substance Use Disorders (SU/SUD) Among Veterans on LTOT Between January 1, 2014 to December 31, 2019; Fig. 2b: Survival Estimates for Fatal Overdose by Substance Use and Substance Use Disorders (SU/SUD) Among Veterans on LTOT Between January 1, 2014 to December 31, 2019; Fig. 2c: Survival Estimates for Traumatic Deaths by Substance Use and Substance Use Disorders (SU/SUD) Among Veterans on LTOT Between January 1, 2014 to December 31, 2019.

The majority of Veterans (79.5 %) were on LTOT and did not have additional SU/SUD (LTOT-only group) (Fig. 1). Among Veterans with one additional SU/SUD, the most common substance group was alcohol (9.8 %), followed by sedative (8.1 %), cannabis (6.6 %), and stimulant (0.6 %). More complex combinations involving two or more substances or diagnoses were less common, with the most common combssinations being alcohol and cannabis (1.5 %), alcohol and sedatives (1.2 %), and cannabis and sedatives (0.9 %) (Fig. 2).

### 3.3 Prevalence of all-cause mortality and primary causes of deaths

Approximately, 1 out of 7 Veterans (14.2 %, *n* = 23,813 all-cause mortality) died within the follow-up period (Table 2). Mortality was higher among Veterans with alcohol (16.4 %) and sedative SU/SUD (15.0 %) compared to LTOT-only (14.2 %). The prevalence of fatal overdose was significantly higher among those co-using substances compared to the LTOT-only group (χ²=93.76, p < 0.001). Veterans with stimulant SU/SUD had the highest fatal overdose (0.74 %), followed by those with sedative (0.58 %), alcohol (0.48 %), and cannabis SU/SUD (0.24 %). For traumatic deaths, Veterans with stimulant SU/SUD had a higher prevalence of accidents or unintentional injuries (0.53 %) and homicide (0.11 %), while those with sedative SU/SUD had the highest prevalence of suicide (0.47 %) compared to Veterans not using any additional substances. Table 3

**Table 2:** Prevalence of all-cause mortality, fatal overdose, and traumatic deaths among veterans on long-term opioid therapy and multiple substance use and disorders between January 1, 2014, to December 31, 2019 (N = 167,547).

Category (N, %)	Overall N (%)	No SU/SUD (n = 117,864)	**Alcohol**^a^ (n = 16,367)	**Cannabis**^a^ (n = 11,039)	**Sedative**^a^ (n = 13,510)	**Stimulant**^a^ (n = 952)	Chi-square (X^2^), p values
**All-Cause Mortality**	23,813 (14.20 %)	17,868 (15.16 %)	2685 (16.40 %)	1129 (10.23 %)	2020 (14.95 %)	111 (11.66 %)	**X**^**2**^**= 219.46,** **p < 0.001**
**Fatal Overdose**	463 (0.28 %)	272 (0.23 %)	79 (0.48 %)	27 (0.24 %)	78 (0.58 %)	7 (0.74 %)	**X**^**2**^**= 93.76,** **p < 0.001**
**Traumatic Deaths**^b^	1103 (0.66 %)	763 (0.65 %)	138 (0.84 %)	63 (0.57 %)	131 (0.97 %)	8 (0.84 %)	**X**^**2**^**= 29.95,** **p < 0.001**
Accidents/Unintentional Injuries	691 (0.41 %)	493 (0.42 %)	90 (0.55 %)	40 (0.36 %)	63 (0.47 %)	5 (0.53 %)	
Suicide	376 (0.22 %)	253 (0.21 %)	38 (0.23 %)	19 (0.17 %)	64 (0.47 %)	2 (0.21 %)	
Homicide	36 (0.02 %)	17 (0.01 %)	10 (0.06 %)	4 (0.04 %)	4 (0.03 %)	1 (0.11 %)	

SU/SUD =  Substance use or substance use disorder

a One additional SU/SUD.

b The chi-square (X2) test was performed only for overall traumatic deaths. Chi-square tests were not conducted for specific types of traumatic deaths due to small numbers in some subgroups.

**Table 3:** Survival analysis for all-cause mortality (23,813), fatal overdose (n = 463), and traumatic deaths (n = 1103) among Veterans on long-term opioid therapy (LTOT) adjusted for demographics, smoking status, psychoactive drugs, care assessment needs score, and hospitalization within one year between January 1, 2014, to December 31, 2019.

**LTOT Categories**	**All-cause mortality** (HR [95 % CI], p-value)^a^	**Fatal overdose** (HR [95 % CI], p-value)^b^	**Traumatic deaths** (HR [95 % CI], p-value)^c^
No SU/SUD	(reference)	(reference)	(reference)
Alcohol^d^	1.06 (0.99, 1.14), p = 0.073	**1.43 (1.10, 1.86), p = 0.008**	1.15 (0.96, 1.39), p = 0.140
Cannabis^d^	**1.16 (1.03, 1.30), p = 0.018**	1.09 (0.71, 1.65), p = 0.700	1.10 (0.85, 1.42), p = 0.484
Sedative^d^	**1.29 (1.10, 1.52), p = 0.002**	**1.40 (1.04 1.91), p = 0.029**	**1.30 (1.05, 1.61), p = 0.017**
Stimulant^d^	**1.54 (1.17, 2.02), p = 0.002**	**3.29 (1.60, 6.77), p = 0.003**	1.55 (0.76, 3.17), p = 0.229

HR =  Hazard ratio; CI =  Confidence intervals; SU/SUD =  Substance use or substance use disorder

Notes: Results from competing risks model where non-overdose and non-traumatic deaths were treated as competing risks.

a All-cause mortality model included time-varying covariates to address violations of the Cox proportional hazards assumptions

b Non-overdose deaths were treated as competing risks

c Non-traumatic deaths were treated as competing risks

d One additional SU/SUD.

### 3.4 Survival analysis of all-cause mortality, fatal overdose, and traumatic deaths

In the survival analysis, Veterans with cannabis (hazard ratio [HR]=1.16, 95 % CI=1.03, 1.30, p = 0.018), sedative (HR=1.29, 95 % CI=1.10, 1.52, p = 0.002), and stimulant SU/SUD (HR=1.54, 95 % CI=1.17, 2.02, p = 0.002) showed higher hazard rates of all-cause mortality compared to the LTOT-only group. For fatal overdose, Veterans with alcohol (HR=1.43, 95 % CI=1.10, 1.86, p = 0.008) and sedative SU/SUD (HR=1.40, 95 % CI=1.04, 1.91, p = 0.029) had a nearly 40 % higher hazard rate, while those with stimulant SU/SUD had more than triple the hazard rate (HR=3.29, 95 % CI=1.60, 6.77, p = 0.003) relative to the LTOT-only group. Additionally, Veterans with sedative SU/SUD showed a higher hazard rate of traumatic death compared to LTOT-only (HR=1.30, 95 % CI=1.05, 1.61, p = 0.017).

## 4 Discussion

This study addresses important gaps in the literature regarding substance co-use with opioids among Veterans on LTOT. Despite VA guidelines discouraging opioid use in patients with substance use disorders (SUDs) (VA/VA/DoD Clinical Practice Guideline, T. L. G., 2022), we found one in four Veterans on LTOT either co-used one additional substance detectable by urine drug screen (UDS), reported risky alcohol consumption, or had a diagnosis of a SUD. Among these other substances, alcohol and sedative SU/SUD was the most common, followed by cannabis. Consistent with prior studies (Hawkins et al., 2019, National Academies of Sciences, Engineering, and Medicine, 2025, Park et al., 2015), sedative SU/SUD was associated with higher mortality rates among Veterans on LTOT for all three outcomes, while Veterans with alcohol SU/SUD exhibited an increased mortality rate for fatal overdose relative to Veterans on LTOT who did not use other substances. Moreover, we found that cannabis and stimulant SU/SUD were associated with high rates of all-cause mortality, which to our knowledge is a novel finding specific to Veterans on LTOT. Cannabis use was associated with a 16 % increase in all-cause mortality rate, while stimulant use was associated with a 54 % increase in all-cause mortality rate and a threefold higher rate for fatal overdose.

While previous studies suggest that clinicians are more likely to discontinue opioids among Veterans with SUD (Lovejoy et al., 2017, Wyse et al., 2018), the high prevalence of substance co-use among Veterans on LTOT highlights the need for enhanced screening and harm reduction interventions to address co-occurring substance use patterns. This statistic is particularly concerning because Veterans on LTOT with a diagnosed SUD are more likely to follow riskier opioid trajectories (Axson et al., 2024), increasing their risk for developing opioid use disorder (OUD). We also found that 7.4 % of Veterans on LTOT had an OUD diagnosis, which is lower than prior estimates suggesting that 41.3 % of Veterans on LTOT met criteria for lifetime OUD (Boscarino et al., 2015). This difference is likely due to our study’s focus on OUD diagnoses within the past two years rather than a lifetime prevalence, as well as the exclusion of Veterans receiving treatment for OUD. While LTOT prescribing has declined, over 50 % of Veterans prescribed opioids remain on LTOT (U.S. Department of Veterans Affairs, 2023). Given the risks of tapering LTOT, including overdose and mental health crises (Agnoli et al., 2021) and the VA guidelines of addressing OUD before tapering, pain management should emphasize a patient-centered approach and shared decision-making to balance between risks and benefits of continued LTOT or tapering to discontinuation (Himstreet et al., 2023).

The high prevalence of cannabis SU/SUD among Veterans on LTOT in our study is likely influenced by the increasing accessibility of cannabis due to the legalization of medical and recreational use in many states (Schaeffer, 2024). Many patients now use cannabis for pain management (Campbell et al., 2018), and some Veterans express interest in, or are actively using, cannabis to replace opioids (Nevedal et al., 2023). However, both the VA and Centers for Disease Control and Prevention (CDC) offer limited guidance on prescribing cannabis for pain management and the approach providers should take to co-use of cannabis and prescription opioids. Moreover, there is a lack of evidence that cannabis is effective on managing pain, either as an adjunct or replacement to LTOT. For example, one study found that cannabis use was not associated with improved pain interference in Veterans on LTOT (Becker et al., 2022). Furthermore, combining cannabis with opioids may increase short-term mortality risk, especially among older Veterans (Keyhani et al., 2022b).

Stimulant SU/SUD among Veterans on LTOT is associated with significantly higher fatal overdose rates, which were three times higher than among Veterans with LTOT-only in our study. This finding aligns with national trends, which show a dramatic rise in opioid-involved stimulant overdoses–quadrupled between 2012 and 2018–and often involving heroin as well as synthetic and natural opioids (Coughlin et al., 2022). Similarly, another study conducted in the general population reported a twofold increase in the hazard of fatal overdose for individuals combining stimulants with opioids compared to opioids alone (Palis et al., 2022). While current VA guidelines recommend avoiding LTOT in patients with known SUD and advise against concurrent alcohol or benzodiazepine use (VA/VA/DoD Clinical Practice Guideline, T. L. G., 2022), they do not provide specific recommendations for regular screening of substances like cannabis and stimulants. Expanding these guidelines to include routine screening for a broader range of substances is particularly urgent given the growing prevalence of cannabis use among Veterans on LTOT and the significant association between stimulant co-use and overdose.

In addition to cannabis and stimulants, our study confirmed the well-documented associations of alcohol and sedative SU/SUD in combination with opioids. Alcohol SU/SUD increased the rate of fatal overdose by 35 %, while sedative SU/SUD doubled the fatal overdose rate and was associated with a 30 % higher rate of traumatic deaths. These findings are consistent with prior research showing that co-prescription of benzodiazepines is associated with a higher mortality rates than opioids alone (Gaither et al., 2016b, Gaither et al., 2016a). Furthermore, hazardous alcohol consumption among LTOT patients is often under-addressed, as these patients are less likely to receive alcohol-related interventions compared to those not on opioids (Joudrey et al., 2024). Veterans who co-use alcohol and opioids experience disproportionately high fatal overdose rates, with a particularly high prevalence among Black and Hispanic Veterans (Lin, Bonar, et al., 2021). One study conducted in the general population found that individuals who co-use alcohol and opioids had a fatal opioid overdose rate of approximately 15 %, while those who co-use benzodiazepines with opioids had a rate exceeding 20 % in 2017 (Tori et al., 2020). Although not specific to overdose-related deaths, another study found that individuals who co-use sedatives and opioids had a 20 % higher likelihood of experiencing an opioid overdose than those using opioids alone (Cho et al., 2020).

Given the high prevalence of SUSUD among Veterans and the association between additional substance use and overdose, our findings suggest an urgent need to expand guidelines to include routine screening for a broader range of substances and substance use disorders. While risk mitigation strategies like urine drug screening (UDS) are widely utilized (Sandbrink et al., 2020), there is variability in their implementation across VA facilities. This variation is influenced by differences in provider practices and patient preferences and lack of conclusive evidence supporting uniform protocols (VA/VA/DoD Clinical Practice Guideline, T. L. G., 2022). These inconsistencies may hinder clinicians’ ability to standardize monitoring and adequately address the full spectrum of overdose risks. Clearer and more actionable guidance is needed, with specific recommendations on screening frequency, methods (biomarkers, self-report, or both), and follow-up interventions. Expanding and standardizing these screening protocols would enable earlier identification of high-risk behaviors, empowering clinicians to implement harm reduction measures tailored to the individual. Such measures could include tapering LTOT, increasing patient monitoring, and providing tailored substance use treatment. Future research should prioritize evaluating the effectiveness of these expanded protocols in reducing overdose risks, as well as assessing their feasibility and acceptability in clinical practice.

Since overdose risk is also shaped by physiological, psychological, and socio-structural factors, expanding harm reduction efforts should include services such as naloxone distribution, medications for opioid use disorder (MOUD), and holistic and complementary approaches (Bennett et al., 2022). Additionally, peer-delivered interventions led by Veterans with lived experience of substance use may mitigate barriers to overdose prevention (Ranney et al., 2024), particularly for socially isolated Veterans or those reluctant to engage with traditional healthcare services. Community-based care models are also important for Veterans who are not connected to VA, as nearly one-fifth of VA mental health patients receive community-based services that address major social determinants of health and co-occurring substance use disorders (Bhalla et al., 2020).

### 4.1 Limitations

Our findings are subject to certain caveats. Although urine drug screens (UDS) provide objective substance use data, they may underestimate SU/SUD prevalence due intermittent use and co-use that go undetected, and 10 % of Veterans on LTOT may not have provided a UDS (Sandbrink et al., 2020). Additionally, the lack of UDS frequency data per person limits assessment of screening consistency. We did not categorize opioid UDS results as consistent or aberrant relative to prescriptions or stratify by opioid dose (e.g., low-dose oxycodone), and thus our findings do not speak to issues of prescription adherence and potential diversion. We excluded Morphine Equivalent Daily Dose (MEDD) as an exposure due to extreme skewness from large methadone prescriptions, limiting dose-related risk assessment. There is likely under-assessment of SU/SUD. We also excluded patients prescribed buprenorphine due to its primary use for opioid use disorder, limiting generalizability to those treated with buprenorphine for chronic pain. We used a retrospective design, excluded patients in treatment for opioid use, and did not have measures for community substance use treatment outside the VA system, all of which affect documentation of SU/SUD in VA medical records. Our use of ICD-coded SUD diagnoses from the prior two years does not distinguish between active and past conditions, potentially misclassifying SUD status and underestimating active cases.

For alcohol use, VA clinician-administered screenings may underreport hazardous alcohol use compared to self-reports (Bradley et al., 2011), as ICD-coded AUD diagnoses likely incorporate multiple assessments beyond AUDIT-C, making direct comparisons between screening-based and diagnostic measures challenging. Our classification of sedative SU/SUD relied on UDS-detected benzodiazepines and ICD-coded sedative use disorders, potentially underestimating use. The discrepancy between UDS-positive benzodiazepine use (10.2 %) and prescriptions (18.5 %) suggests variability in adherence, detection sensitivity, or UDS accuracy. Additionally, we could not distinguish prescribed from non-prescribed benzodiazepines. However, given the high prevalence of opioid and benzodiazepine co-prescribing (Sun et al., 2017) and the ongoing misuse of these prescribed medications (Taha et al., 2024), including prescribed benzodiazepines would allow for a more comprehensive assessment of their association with overdose. Our mutually exclusive SU/SUD categorization do not account for the co-occurrent of multiple types of SU/SUD in addition to LTOT, limiting the ability to isolate the effects of individual substances. Finally, our findings are specific to VA-enrolled Veterans, who have distinct demographic and health profiles, and may not generalize to non-Veteran populations.

## 5 Conclusion

Our study highlights the high prevalence of SU/SUD among patients on LTOT and the increased risk of early mortality for patients with co-occurring LTOT and SU/SUD. These findings support the need for comprehensive and systematic screening within VA to address co-occurring SU/SUD in this population. Healthcare providers and policymakers should consider incorporating routine screening for a broader range of substances and adopting evidence-based approaches to intervention and harm reduction. Future research should focus on evaluating standardized screening protocols and identifying tailored strategies to reduce overdose risk for patients on LTOT with SU/SUD. These efforts are crucial to improving outcomes for Veterans on LTOT and ensuring equitable, patient-centered care.

## CRediT authorship contribution statement

**Salomeh Keyhani:** Writing – review & editing, Validation, Supervision, Funding acquisition. **Thye Peng Ngo:** Writing – review & editing, Writing – original draft, Methodology, Formal analysis, Conceptualization. **Hoggatt Katherine J:** Writing – review & editing, Validation, Supervision. **Samuel Leonard:** Writing – review & editing, Validation, Data curation.

## Author disclosures

This work was supported by VA Health Services Research and Development IIR grant 18–231–2 from the US Department of Veterans Affairs Health Services. Support for US Department of Veterans Affairs (VA) and Centers for Medicare & Medicaid Services data was provided by the VA, VA Health Services Research and Development Service, and VA Information Resource Center (project numbers SDR 02–237 and 98–004)

## Declaration of Competing Interest

The authors declare that they have no known competing financial interests or personal relationships that could have appeared to influence the work reported in this paper.
