# Supplementary material for: Substance use and use disorders among Veterans on long-term opioid therapy
Source: Drug Alcohol Depend Rep. 2025 May 19;16:100347. doi: 10.1016/j.dadr.2025.100347 (PMC12166433; doi:10.1016/j.dadr.2025.100347)
Supplement: Supplementary file 1 — Supplementary material [file mmc1.docx]

**Supplemental:** ICD-9 and 10 Codes

| **Category** | **ICD-9** | **ICD-10** |
| --- | --- | --- |
| **Mental health diagnoses** | | |
| Psychosis | 295.00-295.04,  295.10-295.14,  295.20-295.24,  295.30-295.34,  295.40-295.44,  295.50-295.54,  295.60-295.64,  295.70-295.74,  295.80-295.84,  295.90-295.94,  297.0-297.9, 298.0-298.9 | F20-25, F28, F29, F53.1 |
| Depression | 293.83, 296.20, 296.21, 296.22, 296.23, 296.24, 296.30, 296.31, 296.32, 296.33, 296.34, 296.82, 300.4, 311 | F06.31, F06.32, F32.0, F32.1, F32.2, F32.3, F32.8, F32.89, F32.9, F33.0, F33.1, F33.2, F33.3, F33.40, F33.8, F33.9 |
| Bipolar disorder | 296.10, 296.11, 296.12, 296.13, 296.14, 296.40, 296.41, 296.42, 296.43, 296.44, 296.50, 296.51, 296.52, 296.53, 296.54, 296.60, 296.61, 296.62, 296.63, 296.64, 296.7, 296.80, 296.81, 296.82, 296.89 | F31.0, F31.10, F31.11, F31.12, F31.13, F31.2, F31.30, F31.31, F31.32, F31.4, F31.5, F31.60, F31.61, F31.62, F31.63, F31.64, F31.70, F31.81, F31.89, F31.9 |
| PTSD | 309.82 | F43.10, F43.11, F43.12 |
| Anxiety | 293.84, 300.00, 300.01, 300.02, 300.09, 300.20, 300.21, 300.22, 300.23, 300.29, 300.3 | F06.4, F40.00, F40.01, F40.02, F40.10, F40.11, F40.210, F40.218, F40.220, F40.228, F40.230, F40.231, F40.232, F40.233, F40.240, F40.241, F40.242, F40.243, F40.248, F40.290, F40.291, F40.298, F40.8, F40.9, F41, F41.0, F41.1, F41.3, F41.8, F41.9, F42, F42.2, F42.3, F42.4, F42.8, F42.9, F45.20, F45.21, F45.22, F45.29 |
| Self-harm | E950-E959 | T36-T57, T59-T65, T71  X71-X84 |
| **Pain-related diagnoses** | | |
| Back and spine disorders | 349.39, 353.1, 353.3, 353.4, 720, 720.0, 720.1, 720.2, 720.8, 720.81, 720.89, 720.9, 721.2, 721.3, 721.4, 721.41, 721.42, 721.5, 721.6, 721.7, 721.8, 721.9, 721.90, 721.91, 722, 722.0, 722.1, 722.10, 722.11, 722.2, 722.3, 722.30, 722.31, 722.32, 722.39, 722.5, 722.51, 722.52, 722.6, 722.7, 722.70, 722.72, 722.73, 722.8, 722.80, 722.82, 722.83, 722.9, 722.90, 722.92, 722.93, 724, 724.0, 724.00, 724.01, 724.02, 724.03, 724.09, 724.1, 724.2, 724.3, 724.4, 724.5, 724.6, 724.7, 724.70, 724.71, 724.79, 724.8, 724.9, 738.3, 738.4, 738.5, 738.6, 739.2, 739.3, 739.4, 739.5, 756, 756.1, 756.11, 756.12, 756.13, 756.15, 756.16, 756.17, 756.19, 839.2, 839.20, 839.21, 839.4, 839.40, 839.41, 839.42, 839.49, 846, 846.0, 846.1, 846.2, 846.3, 846.8, 846.9, 847.1, 847.2, 847.3, 847.4, 847.9, 848.5 | G54.1, G54.3, G54.4, G96.11, L40.53, M08.1, M25.78, M43, M43.0, M43.00, M43.04, M43.05, M43.06, M43.07, M43.08, M43.09, M43.1, M43.10, M43.14, M43.15, M43.16, M43.17, M43.18, M43.19, M43.5X, M43.5X4, M43.5X5, M43.5X6, M43.5X7, M43.5X8, M43.5X9, M43.6, M43.8, M43.8X, M43.8X4, M43.8X5, M43.8X6, M43.8X7, M43.8X8, M43.8X9, M43.9, M45, M45.0, M45.4, M45.5, M45.6, M45.7, M45.8, M45.9, M46, M46.0, M46.00, M46.04, M46.05, M46.06, M46.07, M46.08, M46.09, M46.1, M46.2, M46.4, M46.40, M46.44, M46.45, M46.46, M46.47, M46.48, M46.49, M46.8, M46.80, M46.84, M46.85, M46.86, M46.87, M46.88, M46.89, M46.9, M46.90, M46.94, M46.95, M46.96, M46.97, M46.98, M46.99, M47, M47.10, M47.14, M47.15, M47.16, M47.2, M47.20, M47.24, M47.25, M47.26, M47.27, M47.28, M47.8, M47.814, M47.815, M47.816, M47.817, M47.818, M47.819, M47.89, M47.894, M47.895, M47.896, M47.897, M47.898, M47.899, M47.9, M48, M48.0, M48.00, M48.04, M48.05, M48.06, M48.061, M48.062, M48.07, M48.08, M48.10, M48.14, M48.15, M48.16, M48.17, M48.18, M48.19, M48.2, M48.20, M48.24, M48.25, M48.26, M48.27, M48.3, M48.30, M48.34, M48.35, M48.36, M48.37, M48.38, M48.8, M48.8X, M48.8X4, M48.8X5, M48.8X6, M48.8X7, M48.8X8, M48.8X9, M48.9, M49, M49.84, M49.85, M49.86, M49.87, M49.88, M49.89, M51, M51.04, M51.05, M51.06, M51.1, M51.14, M51.15, M51.16, M51.17, M51.2, M51.24, M51.25, M51.26, M51.27, M51.3, M51.34, M51.35, M51.36, M51.37, M51.4, M51.44, M51.45, M51.46, M51.47, M51.8, M51.84, M51.85, M51.86, M51.87, M51.9, M53, M53.0, M53.1, M53.2, M53.2X, M53.2X4, M53.2X5, M53.2X6, M53.2X7, M53.2X8, M53.2X9, M53.3, M53.8, M53.84, M53.85, M53.86, M53.87, M53.88, M53.9, M54, M54.0, M54.00, M54.04, M54.05, M54.06, M54.07, M54.08, M54.09, M54.1, M54.10, M54.14, M54.15, M54.16, M54.17, M54.18, M54.3, M54.30, M54.31, M54.32, M54.4, M54.40, M54.41, M54.42, M54.5, M54.6, M54.8, M54.89, M54.9, M62.830, M95.4, M95.5, M96.1, M99, M99.0, M99.02, M99.03, M99.04, M99.05, M99.1, M99.12, M99.14, M99.18, M99.5, M99.53, M99.7, M99.73, M99.79, M99.8, M99.82, M99.83, M99.84, M99.85, M99.88, Q67.5, Q76.0, Q76, Q76.0, Q76.1, Q76.2, Q76.3, Q76.4, Q76.41, Q76.414, Q76.415, Q76.419, Q76.49, S23, S23.100, S23.100A, S23.101, S23.101A, S23.101D, S23.101S, S23.111, S23.111A, S23.111D, S23.111S, S23.121, S23.121A, S23.121D, S23.121S, S23.123, S23.123A, S23.123D, S23.123S, S23.131, S23.131A, S23.131D, S23.131S, S23.133, S23.133A, S23.133D, S23.133S, S23.141, S23.141A, S23.141D, S23.141S, S23.143, S23.143A, S23.143D, S23.143S, S23.151, S23.151A, S23.151D, S23.151S, S23.153, S23.153A, S23.153D, S23.153S, S23.161, S23.161A, S23.161D, S23.161S, S23.163, S23.163A, S23.163D, S23.163S, S23.171, S23.171A, S23.171D, S23.171S, S23.2, S23.20, S23.20XA, S23.20XD, S23.20XS, S23.29, S23.29XA, S23.29XD, S23.29XS, S23.3, S23.3XXA, S23.3XXD, S23.3XXS, S23.8, S23.8XXA, S23.8XXD, S23.8XXS, S23.9, S23.9XXA, S23.9XXD, S23.9XXS, S29.019, S29.019A, S29.019D, S29.019S, S33, S33.0, S33.0XXA, S33.1, S33.10, S33.100, S33.100A, S33.101, S33.101A, S33.101D, S33.101S, S33.11, S33.111, S33.111A, S33.111D, S33.111S, S33.12, S33.121, S33.121A, S33.121D, S33.121S, S33.13, S33.131, S33.131A, S33.131D, S33.131S, S33.14, S33.140, S33.140A, S33.140D, S33.141, S33.141A, S33.141D, S33.141S, S33.2, S33.2XXA, S33.2XXD, S33.2XXS, S33.3, S33.30, S33.30XA, S33.30XD, S33.30XS, S33.39, S33.39XA, S33.39XD, S33.39XS, S33.5, S33.5XXA, S33.5XXD, S33.5XXS, S33.6, S33.6XXA, S33.6XXD, S33.6XXS, S33.8, S33.8XXA, S33.8XXD, S33.8XXS, S33.9, S33.9XXA, S33.9XXD, S33.9XXS, S39.012, S39.012A, S39.012D, S39.012S, S39.92 |
| Neck and spine disorders | 353.2, 721, 721.0, 721.1, 722, 722.0, 722.4, 722.71, 722.81, 722.91, 723.0, 723.1, 723.2, 723.3, 723.4, 723.5, 723.6, 723.7, 723.8, 723.9, 738.2, 739.1, 839.00, 839.01, 839.02, 839.03, 839.04, 839.05, 839.06, 839.07, 839.08, 847.0, 848.2 | G54.2, M43, M43.0, M43.00, M43.01, M43.02, M43.03, M43.1, M43.11, M43.12, M43.13, M43.3, M43.4, M43.5, M43.5X, M43.5X2, M43.5X3, M43.5X4, M43.6, M43.8, M43.8X, M43.8X1, M43.8X2, M43.8X3, M45.1, M45.2, M45.3, M46, M46.0, M46.01, M46.02, M46.03, M46.4, M46.41, M46.42, M46.43, M46.8, M46.81, M46.82, M46.83, M46.9, M46.91, M46.92, M46.93, M47, M47.1, M47.11, M47.12, M47.13, M47.2, M47.22, M47.8, M47.81, M47.811, M47.812, M47.813, M47.89, M47.891, M47.892, M47.893, M48, M48.0, M48.01, M48.02, M48.03, M48.1, M48.11, M48.12, M48.13, M48.2, M48.21, M48.22, M48.23, M48.3, M48.31, M48.32, M48.33, M48.8, M48.8X, M48.8X1, M48.8X2, M48.8X3, M49.81, M49.82, M49.83, M50, M50.0, M50.00, M50.01, M50.02, M50.020, M50.021, M50.022, M50.023, M50.03, M50.1, M50.10, M50.11, M50.12, M50.120, M50.121, M50.122, M50.123, M50.13, M50.2, M50.20, M50.21, M50.22, M50.220, M50.221, M50.222, M50.223, M50.23, M50.3, M50.30, M50.31, M50.32, M50.320, M50.321, M50.322, M50.323, M50.33, M50.8, M50.80, M50.81, M50.82, M50.820, M50.821, M50.822, M50.823, M50.83, M50.9, M50.90, M50.91, M50.92, M50.920, M50.921, M50.922, M50.923, M50.93, M53, M53.0, M53.1, M53.2, M53.2X, M53.2X1, M53.2X2, M53.2X3, M53.8, M53.81, M53.82, M53.83, M54, M54.0, M54.01, M54.02, M54.03, M54.09, M54.1, M54.11, M54.12, M54.13, M54.2, M95.3, M99, M99.0, M99.01, M99.1, M99.11, M99.3, M99.31, M99.5, M99.51, M99.6, M99.61, M99.7, M99.71, M99.8, M99.81, Q76.41, Q76.411, Q76.412, Q76.413, S13, S13.0, S13.0XXA, S13.1, S13.10, S13.100, S13.100A, S13.100D, S13.101, S13.101A, S13.101D, S13.101S, S13.11, S13.111, S13.111A, S13.111D, S13.111S, S13.12, S13.120, S13.120A, S13.121, S13.121A, S13.121D, S13.121S, S13.13, S13.130, S13.130A, S13.131, S13.131A, S13.131D, S13.131S, S13.14, S13.140, S13.140A, S13.141, S13.141A, S13.141D, S13.141S, S13.15, S13.150, S13.150A, S13.151, S13.151A, S13.151D, S13.151S, S13.16, S13.160, S13.160A, S13.160D, S13.161, S13.161A, S13.161D, S13.161S, S13.17, S13.170, S13.170A, S13.170D, S13.171, S13.171A, S13.171D, S13.171S, S13.18, S13.180, S13.180A, S13.181, S13.181A, S13.181D, S13.181S, S13.2, S13.20, S13.20XA, S13.20XD, S13.20XS, S13.29, S13.29XA, S13.29XD, S13.29XS, S13.4, S13.4XXA, S13.4XXD, S13.4XXS, S13.5, S13.5XXA, S13.8, S13.8XXA, S13.8XXD, S13.8XXS, S13.9, S13.9XXA, S13.9XXD, S13.9XXS, S16.1, S16.1XXA, S16.1XXD, S16.1XXS |
| Osteoarthritis | 715, 715.0, 715.00, 715.04, 715.09, 715.1, 715.10, 715.11, 715.12, 715.13, 715.14, 715.15, 715.16, 715.17, 715.18, 715.2, 715.20, 715.21, 715.22, 715.23, 715.24, 715.25, 715.26, 715.27, 715.28, 715.3, 715.30, 715.31, 715.32, 715.33, 715.34, 715.35, 715.36, 715.37, 715.38, 715.8, 715.80, 715.89, 715.9, 715.90, 715.91, 715.92, 715.93, 715.94, 715.95, 715.96, 715.97, 715.98 | M15, M15.0, M15.1, M15.2, M15.3, M15.4, M15.8, M15.9, M16, M16.0, M16.1, M16.10, M16.11, M16.12, M16.2, M16.3, M16.30, M16.31, M16.32, M16.4, M16.5, M16.50, M16.51, M16.52, M16.6, M16.7, M16.9, M17, M17.0, M17.1, M17.10, M17.11, M17.12, M17.2, M17.3, M17.30, M17.31, M17.32, M17.4, M17.5, M17.9, M18, M18.0, M18.1, M18.10, M18.11, M18.12, M18.2, M18.3, M18.30, M18.31, M18.32, M18.4, M18.5, M18.50, M18.51, M18.52, M18.9, M19, M19.0, M19.01, M19.011, M19.012, M19.019, M19.02, M19.021, M19.022, M19.029, M19.03, M19.031, M19.032, M19.039, M19.04, M19.041, M19.042, M19.049, M19.07, M19.071, M19.072, M19.079, M19.1, M19.11, M19.111, M19.112, M19.119, M19.12, M19.121, M19.122, M19.129, M19.13, M19.131, M19.132, M19.139, M19.14, M19.141, M19.142, M19.149, M19.17, M19.171, M19.172, M19.179, M19.2, M19.21, M19.211, M19.212, M19.219, M19.22, M19.221, M19.222, M19.229, M19.23, M19.231, M19.232, M19.239, M19.24, M19.241, M19.242, M19.249, M19.27, M19.271, M19.272, M19.279, M19.9, M19.90, M19.91, M19.92, M19.93, M24.7 |
| Neuropathy | 250.6, 250.60, 250.61, 250.62, 250.63, 053.12, 053.13, 337.00, 337.09, 337.1, 350, 350.1, 350.2, 350.8, 350.9, 352.1, 353.8, 353.9, 354.8, 354.9, 355.79, 355.8, 355.9, 356.4, 356.8, 356.9, 357.2, 729.2 | B02.22, B02.23, E08.42, E10.40, E10.41, E10.42, E10.43, E10.610, E11.40, E11.41, E11.42, E11.43, E11.610, E13.40, E13.41, E13.42, E13.43, E13.610, G50.0, G50.8, G50.9, G52.1, G54.8, G54.9, G56.8, G56.80, G56.81, G56.82, G56.83, G56.9, G56.90, G56.91, G56.92, G56.93, G57.8, G57.80, G57.81, G57.82, G57.83, G57.9, G57.90, G57.91, G57.92, G57.93, G58.7, G58.8, G58.9, G59, G60, G60.2, G60.3, G60.8, G60.9, G61.9, G62, G62.0, G62.89, G62.9, G63, G90.09, G99.0, M79.2 |
| Headache | 307.81, 339, 339.0, 339.00, 339.01, 339.02, 339.03, 339.04, 339.05, 339.09, 339.1, 339.10, 339.11, 339.12, 339.2, 339.20, 339.21, 339.22, 339.4, 339.41, 339.42, 339.43, 339.44, 339.8, 339.81, 339.82, 339.83, 339.84, 339.85, 339.89, 346, 346.0, 346.00, 346.01, 346.02, 346.03, 346.1, 346.10, 346.11, 346.12, 346.13, 346.2, 346.20, 346.21, 346.22, 346.23, 346.3, 346.30, 346.31, 346.32, 346.33, 346.4, 346.40, 346.41, 346.42, 346.43, 346.5, 346.50, 346.51, 346.52, 346.53, 346.6, 346.60, 346.61, 346.62, 346.63, 346.7, 346.70, 346.71, 346.72, 346.73, 346.8, 346.80, 346.81, 346.82, 346.83, 346.9, 346.90, 346.91, 346.92, 346.93, 784.0 | G43, G43.0, G43.00, G43.001, G43.009, G43.01, G43.011, G43.019, G43.1, G43.10, G43.101, G43.109, G43.11, G43.111, G43.119, G43.4, G43.40, G43.401, G43.409, G43.41, G43.411, G43.419, G43.5, G43.50, G43.501, G43.509, G43.51, G43.511, G43.519, G43.6, G43.60, G43.601, G43.609, G43.61, G43.611, G43.619, G43.7, G43.70, G43.701, G43.709, G43.71, G43.711, G43.719, G43.B, G43.B0, G43.B1, G43.C, G43.C0, G43.C1, G43.80, G43.801, G43.809, G43.81, G43.811, G43.819, G43.82, G43.821, G43.829, G43.83, G43.831, G43.839, G43.9, G43.90, G43.901, G43.909, G43.91, G43.911, G43.919, G44, G44.0, G44.00, G44.001, G44.009, G44.01, G44.011, G44.019, G44.02, G44.021, G44.029, G44.03, G44.031, G44.039, G44.04, G44.041, G44.049, G44.05, G44.051, G44.059, G44.09, G44.091, G44.099, G44.1, G44.2, G44.20, G44.201, G44.209, G44.21, G44.211, G44.219, G44.22, G44.221, G44.229, G44.3, G44.30, G44.301, G44.309, G44.31, G44.311, G44.319, G44.32, G44.321, G44.329, G44.5, G44.51, G44.52, G44.53, G44.59, G44.8, G44.81, G44.82, G44.83, G44.84, G44.85, G44.89, M54.81, R51 |
| Traumatic brain injury | 310.2 | F07.81 |
| **Social-related factors** | | |
| Homelessness | V60.0 | Z59.0 |
| Lack of social support | V60.3, V62.4 | Z60.2, Z60.4 |
| **Substance use disorders** | | |
| Alcohol use disorder | 303.0, 303.9, 305.0 | F10 |
| Cannabis use disorder | 304.3, 305.2 | F12 |
| Cocaine use disorder | 304.2, 305.6 | F14 |
| Opioid use disorder | 304.0, 304.7, 305.5 | F11 |
| Sedative use disorder | 304.1, 305.4 | F13 |
| Stimulant use disorder | 304.4, 305.7 | F15 |
| Other drug use disorder | 304.5, 304.6, 304.8, 304.9, 305.3, 305.8, 305.9 | F16, F18, F19 |
| **Mortality** | | |
| Fatal overdose |  | T36–T50, X40–X44, X60–X64, Y10–Y14 |
| Traumatic deaths |  |  |
| Accidents/unintentional injury |  | V01–X59 |
| Suicide |  | X60–X84 |
| Homicide |  | X85–Y09 |
